# High pathogenicity avian influenza (HPAI) H5N1 virus detected in brown Skua using portable laboratory while at sea in Antarctica

**DOI:** 10.1128/mra.00041-25

**Published:** 2025-04-17

**Authors:** Michelle McCulley, Meagan Leanne Dewar, Yee Syuen Low, Andrew Wilson, Ruy Jauregui, Anastasia Chernyavtseva, Joseph O'Keefe

**Affiliations:** 1 Animal Health Laboratory, Diagnostics, Readiness and Surveillance, Biosecurity New Zealand, Ministry for Primary Industries, Upper Hutt, New Zealand; 2 Future Regions Research Centre, Federation University, Melbourne, Australia; Katholieke Universiteit Leuven, Leuven, Belgium

**Keywords:** HPAI, H5N1, Antarctica, portable laboratory, genome analysis, gene sequencing, rapid tests, 48 hours, brown skua

## Abstract

We report the draft genome sequence of a high pathogenicity avian influenza (HPAI) H5N1 clade 2.3.4.4b virus found in a brown skua (*Stercorarius antarcticus*) on Torgersen Island, which was detected and sequenced *in situ* using a portable laboratory while in Antarctica.

## ANNOUNCEMENT

Avian influenza viruses (*Alphainfluenzavirus Orthymyxoviridae*) are classified as low or high pathogenicity (LPAI/HPAI) based on the hemagglutinin (HA) cleavage site sequence (1). HPAIs present a significant threat to human health due to their potential for zoonotic transmission. To improve in-field testing capabilities, a portable laboratory was developed at the Animal Health Laboratory, Ministry for Primary Industries, New Zealand and was deployed onboard the *MV Argus* (IMO: 7104752, Call sign: OVZW2) to Antarctica in December 2024 as part of the EVOKN expedition.

A dead brown skua, exhibiting clinical signs of HPAI, was found on Torgersen Island (64.46^o^S, 64.04^o^W) following a report from the US Antarctic Program (Palmer Station). Oropharyngeal and brain swabs were collected in DNA/RNA shield™ (Zymo Research), with brain swabs collected through the eye socket using a corkscrew. RNA was extracted using the QIAamp Viral RNA Mini kit (Qiagen) on a Bento Lab Pro (Bento Bioworks Ltd). RT-PCR was performed to amplify conserved AIV genome regions (2), using the SuperScript™ III One-Step RT-PCR System with Platinum™ Taq High-Fidelity DNA Polymerase (Thermo Fisher Scientific) on a Bento Lab Pro. Eight AIV segment-specific amplicons were amplified, purified with ExoSAP-IT™ Express (Thermo Fisher Scientific), and quantified using the Qubit 1× dsDNA high sensitivity assay (Thermo Fisher Scientific).

Amplicons were prepared for sequencing using the Rapid Barcoding Kit 24 V14 (SQK-RBK114.24, Oxford Nanopore Technologies [ONT]), using the amplicon sequencing protocol. Pooled libraries of both samples were sequenced using a MinION Mk1B (ONT). Basecalling was performed using Dorado (v7.0.2) with a high-accuracy basecall model. Viral subtype was obtained in real-time using *Flui,* an automated AIV subtyping tool developed in-house (uses the Jensen-Shannon distance to detect similarities between kmer distributions from new reads and reference distributions). Reads from both swabs were pooled and then trimmed using BBDuk v38.84 (3). Pooled reads were mapped with Minimap2 v2.24 to a non-redundant set of 449 AIV sequences (H1-H16 and N1-N9) from GISAID (4). Eight consensus sequences were extracted in Geneious Prime v2021.1.1 (https://www.geneious.com) and then assembled into a single genome. A near-complete genome was generated, with some terminal nucleotides trimmed due to low quality. Sequences were manually polished to bring them in-frame. Assembly statistics are provided in Table 1.

**TABLE 1:** Genome assembly statistics, average depth of coverage, and closest NCBI BLAST hits for each viral genome segment

Genome assembly statistics	
Raw read number	151,387 (74,118 oropharyngeal swabs + 77,269 brain swab)
Number of mapped reads	50,285 (33.2%)
N50 (Kb)	0.829

Segment	Length (bp)	Average depth of coverage	Closest NCBI BLAST match (pairwise ID)
1 - PB2	2,313	254×	PQ129503 (99.2%)
2 - PB1	2,313	144×	PQ114023 (99.5%)
3 - PA	2,209	421×	PQ129502 (99.6%)
4 - HA	1,748	1,928×	PQ114025 (99.3%)
5 - NP	1,497	854×	PQ114018 (99.6%)
6 - NA	1,386	1,212×	PQ300717 (97.0%)
7 - MP	982	2,666×	PQ114076 (99.7%)
8 - NS	769	6,876×	CY020905 (95.4%)

All protocols were performed per manufacturer’s instructions. For software, default parameters were used.

**Fig 1:**
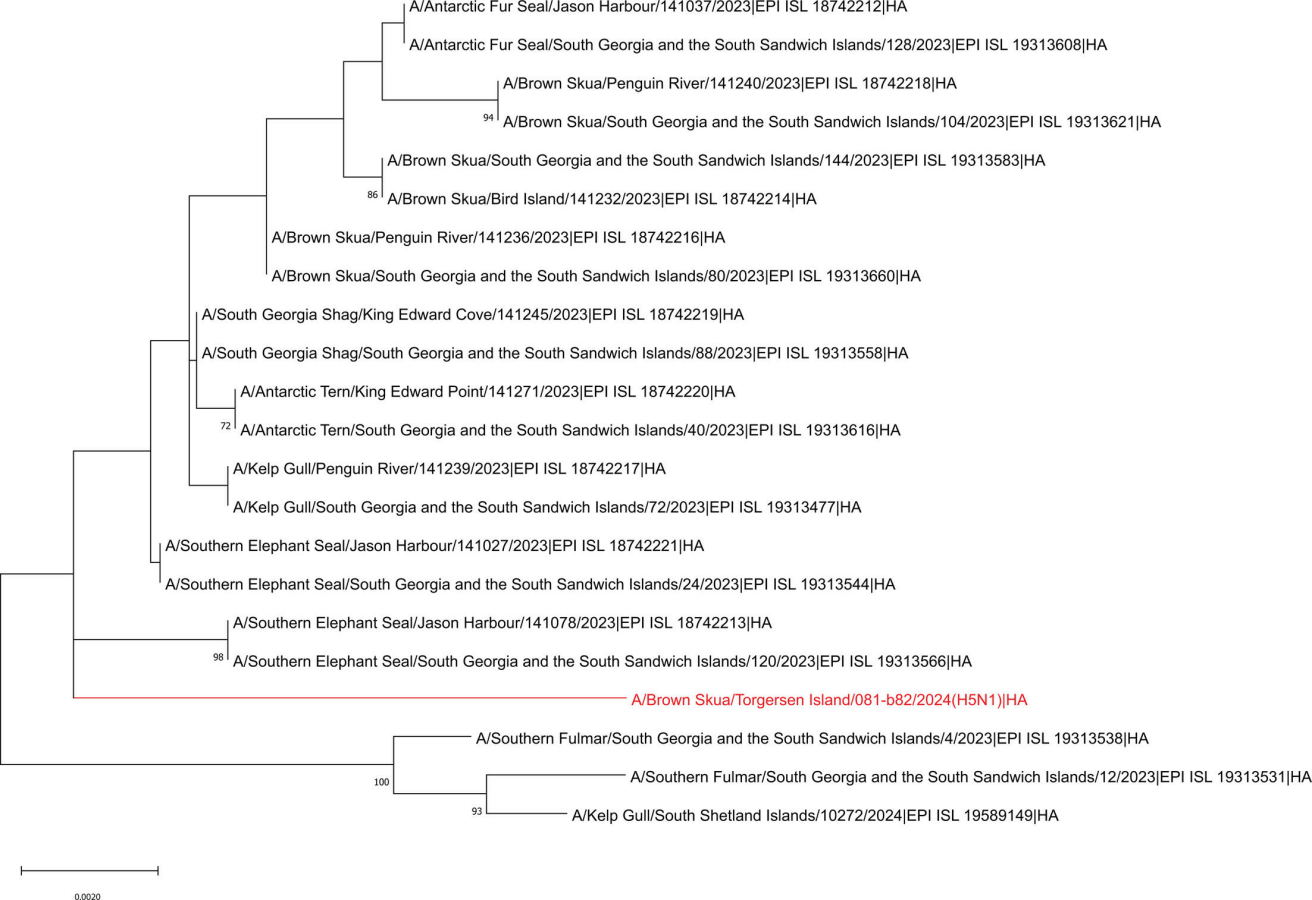
Phylogenetic analysis of a H5N1 HPAI sample taken from a Brown skua on Torgersen Island (red) compared with other publicly available H5N1 clade 2.3.4.4b HPAI genomes from the surrounding Antarctic islands. Reference H5N1 clade 2.3.4.4b HPAI genomes were downloaded from GISAID, followed by extraction of the HA segment for phylogenetic analysis. Alignments were produced using MAFFT v7.490. Evolutionary history was inferred by using the maximum likelihood method and Tamura-Nei model. The tree with the highest log likelihood (−2,808.97) is shown. The percentage of 500 trees in which the associated taxa clustered together is shown next to the branches. Initial trees for the heuristic search were obtained automatically by applying Neighbor-Join and BioNJ algorithms to a matrix of pairwise distances estimated using the Tamura-Nei model and then selecting the topology with a superior log likelihood value. The tree is drawn to scale. This analysis involved 22 nucleotide sequences. There were a total of 1,777 positions in the final data set. Bootstrap values below 70 were removed from the final tree. Evolutionary analyses were conducted in MEGA11 (5).

The consensus HA sequence, extracted from the assembled genome in Geneious Prime v2021.1.1, confirmed a multi-basic cleavage site (PLRERRRKR/GLF) indicative of HPAI per the Offlu Influenza A guide (https://www.offlu.org). All segments closely matched viruses from nearby Antarctic islands, with top NCBI BLAST hits provided in Table 1. Phylogenetic analysis of the consensus HA sequence, and other H5N1 clade 2.3.4.4b HPAI HA sequences detected locally, is shown in Fig. 1.

In summary, we report a high-quality, coding-complete H5N1 clade 2.3.4.4b HPAI genome detected in a brown skua on Torgersen Island. Phylogenetic analysis places this genome among other H5N1 2.3.4.4b HPAI strains from the surrounding Antarctic islands. Sequencing and characterization were completed using a portable laboratory at sea, highlighting the value of in-field testing solutions for avian influenza surveillance in remote locations.

## Data Availability

The data generated for this genome, designated A/Brown Skua/Torgersen Island/o81-b82/2024, have been deposited under Sequence Read Archive BioProject ID PRJNA1210083, GISAID accession EPI_ISL_19645365, and GenBank accessions PV163161-8
